# Labeling of human mesenchymal stem cells with different classes of vital stains: robustness and toxicity

**DOI:** 10.1186/s13287-019-1296-8

**Published:** 2019-06-25

**Authors:** Anna Andrzejewska, Anna Jablonska, Martyna Seta, Sylwia Dabrowska, Piotr Walczak, Miroslaw Janowski, Barbara Lukomska

**Affiliations:** 1 NeuroRepair Department, Mossakowski Medical Research Centre, Polish Academy of Sciences, Warsaw, Poland; 2 Division of MR Research, Russell H. Morgan Department of Radiology and Radiological Science, The Johns Hopkins University School of Medicine, Baltimore, USA

**Keywords:** Mesenchymal stem cells, Cell labeling, Vital stains, Fluorescent dyes, MRI contrast agents

## Abstract

**Background:**

Mesenchymal stem cell (MSC) transplantation has been explored as a new clinical approach to repair injured tissues. However, in order to evaluate the therapeutic activity of MSC, cell tracking techniques are required to determine the fate of transplanted cells in both preclinical and clinical studies. In these aspects, different vital stains offer the potential for labeling and monitoring of grafted cells in vivo*.* It is desirable to have tracking agents which have long-term stability, are not toxic to the cells, and do not affect cell function.

**Methods:**

Here, we selected three different labels: CellTracker™ Green CMFDA, eGFP-mRNA (genetic pre-tag), and Molday ION Rhodamine B™ (nanoparticle-based fluorescent and magnetic label) and performed extensive analysis of their influence on in vitro expansion of human bone marrow-derived mesenchymal stem cells (hBM-MSCs), as well as potential of affecting therapeutic activity and the impact on the durability of staining.

**Results:**

Our study showed that basic hBM-MSC characteristics and functions might be affected by labeling. We observed strong alterations of metabolic activity and morphology after eGFP and CellTracker™ Green CMFDA hBM-MSC staining. Molday ION Rhodamine B™ labeling revealed superior properties relatively to other vital stains. The relative expression level of most of the investigated growth factors remained stable after cell labeling, but we have observed some changes in the case of EGF, GDNF, HGF, and IGF gene expression.

**Conclusions:**

Taken together, we suggest performing similar to ours extensive analysis prior to using any cell label to tag MSC in experiments, as it can thoroughly bias results.

**Electronic supplementary material:**

The online version of this article (10.1186/s13287-019-1296-8) contains supplementary material, which is available to authorized users.

## Background

Stem cell-based regenerative medicine is a new, exciting, and much needed field of research to address the upcoming challenges of aging society and civilization-driven disorders [1]. Mesenchymal stem cells (MSCs) are frequently selected as an optimal source of stem cells for transplantation [2]. It is contingent upon their easy procurement from various sources, efficient expansion, and multitude of applications [3–5]. There are two major modes of stem cell therapeutic activity: direct—replacement of dead cells, and indirect—healing capability through the release of paracrine factors [6]. Particularly, the paracrine mechanisms are appealing as ascribed to the achievement of therapeutic effects even in case of a short life span of transplanted stem cells [7–9]. However, regardless of the mode of action, we are obliged, at least, to deliver stem cells precisely and to be aware of their distribution to become compliant with the current initiative of precision medicine [10].

There are various approaches to pre- and postmortem imaging of transplanted cells with a variable robustness and complexity [11]. The simplest approach is based on pre-transplantation labeling of stem cells with a fluorescent tag followed by a postmortem identification using microscopy. The additional advantage is that cells can be directly spotted in the tissue slices, without the need of additional procedures, which are fraught with a risk of failure, and particularly ineffective if there is uncertainty about the cell location such as after minimally invasive, intravascular routes. The fluorescent tags are particularly suitable for studying primary, inherently highly therapeutic human stem cells such as MSC, as the lengthy process of DNA-based genetic engineering including the process of selection (based on cell sorting or antibiotic exclusion) might age stem cells and affect their native curative properties. There are various classes of labels, which can be used for tagging of stem cells: dyes, nanoparticles, and even mRNA (treated as a smart pre-tag, as it requires the production of fluorescent protein, which then can be detected).

Fluorescent dyes are typically strongly lipophilic, thus rapidly and efficiently tag each type of cells without exception. The easy labeling protocol and vivid fluorescence make them very handy and attractive to study postmortem distribution of transplanted cells [12–14].

The nanoparticle-based methods are also widely used and have their advantages and disadvantages. The possibility for tailoring of the composition of nanoparticles and their decoration with various molecules facilitate their use for multimodal imaging [15]. Particularly, the fluorescent molecules can be easily attached to the various cores, which in addition to postmortem identification of transplanted cells allows for various types of in vivo cellular imaging. The superparamagnetic iron oxide (SPIO) nanoparticles are the earliest, most frequently used method for preclinical and clinical in vivo imaging of stem cells, due to their capability to generate a very high signal in MRI (magnetic resonance imaging) [16–18]. However, the early formulation of non-fluorescent SPIO nanoparticles was negatively affecting chondrogenic differentiation of MSC [19]. The additional potential disadvantage of nanoparticle-based cellular imaging systems is related to the need for the uptake of a sufficient number of nanoparticles, which can be a challenge for non-phagocytic cells. Altogether, the combination of fluorescence and magnetic properties can be a very attractive option for tracking MSC.

Recently the effective production of green fluorescent protein MSC by mRNA-based transfection has been reported, which is a very rapid (hours after transfection), ubiquitous (it labels all cells) and durable (up to 1 week) process and could be also potentially exploited for tagging of stem cells, but it has never been previously studied for this purpose [20]. The advantage of this method could lie in the reporting also on cell viability as the GFP has a short lifetime and can be produced only in living cells due to the need for energy and translational machinery.

While the easiness and robustness of labeling-based methods for post-transplantation stem cell imaging is an advantage, the gradual dilution of label due to the proliferation of transplanted cells and the short lifetime of some labels are weaknesses [21]. Therefore, the pre-transplantation labeling is particularly suitable for the assessment of early distribution of stem cells, which is also a very important task. Moreover, the nanoparticle-based formulations with fluorescent and magnetic components allow not only for postmortem, but also for in vivo imaging, with easiness of postmortem confirmation of cell location [22]. While the success of traditional day-to-day imaging of SPIO-labeled cells is mixed due to difficulties in distinction between transplanted cells and other sources of hypointensities and gradually overtaken with less unambiguous labels such as fluorine, the recent advances with ultra-high speed real-time MRI to image cells during infusion to deploy them in a precise and predictable fashion still make studying SPIO-based nanoparticles highly desired [23, 24].

Here, we selected one label from each of three classes and performed extensive analysis of their influence on in vitro expansion of human MSC, as well as the potential of affecting therapeutic activity and the durability of staining. The choice of labels was based on our previous experience with them and easy achievement of robust labeling, and it includes 5-chloromethylfluorescein diacetate (CellTracker™ Green CMFDA), Molday ION Rhodamine B™ (nanoparticle-based fluorescent and magnetic label), and eGFP-mRNA (genetic pre-tag). While we have previously shown that Molday ION Rhodamine B™ has no negative influence, the study was performed on mouse MSC and included only in vitro expansion and migration, but the process of differentiation and release of paracrine factors has never been studied [25]. Since our research is geared toward clinical translation of therapies based on human bone marrow-derived MSC (hBM-MSC), we have selected these cells as an object of our investigations.

## Methods

### hBM-MSC culture

The hBM-MSCs (Lonza) were thawed and maintained in MSCBM medium supplemented with 10% MCGS (mesenchymal cell growth supplement), l-glutamine, and gentamicin (MSCGM, hMSC SingleQuot Kit, Lonza) in a humidified atmosphere at 37 °C and 5% CO_2_ using 75-cm^2^ flasks for five passages. For the experiments, hBM-MSCs were transferred to 25-cm^2^ flasks (2 × 10^5^ cell/flask) (Thermo Scientific) or 24-well (5 × 10^3^ cell/well) (Thermo Scientific) or 96-well culture plates (1 × 10^3^ cell/well) (Thermo Scientific) and subjected to labeling. The unlabeled hBM-MSC served as a control. The hBM-MSCs used in our study were isolated from 4 healthy, randomly selected adult donors of both sexes aged 19–38.

### Labeling of hBM-MSC with CellTracker™ Green CMFDA

The CellTracker™ Green CMFDA (Life Technologies) labeling processes of hBM-MSC were performed for cells seeded on 96-well (1 × 10^3^ cell/well) plates, 24-well plates (5 × 10^3^cell/well), and 25-cm^2^ bottles (2 × 10^5^ cell/flask). In sterile 15 ml Falcon (Corning Centristar), a mixture of Opti-Mem® I (1×) Reduced Serum Medium (Life Technologies) and 10 μM CellTracker™ Green CMFDA solution in dimethyl sulfoxide (DMSO) (Sigma-Aldrich) was prepared. One microliter of CellTracker™ Green CMFDA stock was added on each 5 ml of Opti-Mem® I (1×) Reduced Serum Medium. Standard growing medium was removed from culture vessel; cells were rinsed twice with phosphate buffer (PBS, Gibco) and placed in a prepared solution of CellTracker™ Green CMFDA and Opti-Mem® I (1×) Reduced Serum Medium. After 40-min incubation at 37 °C, the cells were purified from an excess of reagent by triple PBS rinsing and placed in a medium suitable for further experiments.

### mRNA eGFP transfection of hBM-MSC

The transfection processes of hBM-MSC were performed for cells seeded on 96-well (1 × 10^3^ cell/well) plates, 24-well plates (5 × 10^3^ cell/well), and 25-cm^2^ bottles (2 × 10^5^cell/flask). Data presented in this description are shown for 24-well plates (5 × 10^3^ cell/well). Calculations for different types of vessels were made in proportion to values stated in the text to obtain the final concentration of 0.5 μg/ml mRNA eGFP (Stemgent) in the final transfection mixture. The hBM-MSCs seeded onto 24-well plates were allowed to adhere to the plastic bottoms of the wells overnight. The complexes of eGFP-mRNA and Lipofectamine™ 2000 (Invitrogen, Life Technologies) were performed. Half an hour before transfection, the standard growing medium was removed and cells were rinsed with PBS, then placed in 125 μl of Opti-Mem® I (1×) Reduced Serum Medium. At the same time, 1.19 μl of Lipofectamine® 2000 was diluted with 30.6 μl of Opti-Mem® I (1×) Reduced Serum Medium in a 1.5-ml sterile plastic tube (Eppendorf), followed by 5-min incubation. In a separate tube, mRNA-eGFP was added to the final volume of 31.25 μl in Opti-Mem® I (1×) Reduced Serum Medium. Diluted Lipofectamine® 2000 and mRNA-eGFP were mixed together for lipoplex formation during 20 min at room temperature. The additional 62.5 μl of Opti-Mem® I (1×) Reduced Serum Medium was added to the mixture. At the end, 125 μl/well of the final mixture was introduced to the cells, followed by a 4-h incubation at 37 °C/5% CO_2_, when reaction mix was replaced by standard growing medium.

### Molday ION Rhodamine B™ cell labeling

The SPIO labeling process of hBM-MSC was performed for cells seeded on 96-well (1 × 10^3^ cell/well) plates, 24-well plates (5 × 10^3^cell/well), and 25-cm^2^ bottles (2 × 10^5^cell/flask). hBM-MSCs were incubated with MSCGM™ mesenchymal stem cell growth medium containing 20 μg/ml Molday ION Rhodamine B™ for 18 h (37 °C, 5% CO_2_), then washed with PBS and cultured in MSCGM™ mesenchymal stem cell growth medium.

### Evaluation of cytotoxicity of used labeling methods

The hBM-MSCs were seeded onto 96-well plates at a density of 1 × 10^3^ cells per well and allowed to adhere to the plastic bottoms of the wells overnight. Next day cells were stained with Molday ION Rhodamine B™ and CellTracker™ Green CMFDA or transfected with mRNA eEGFP. One day after the labeling procedure, Cell Counting Kit-8–Cell Proliferation and Cytotoxicity Assay (CCK-8, Dojindo Laboratories) has been used to measure the metabolic activity of hBM-MSC according to the manufacturer instructions. Briefly, 10 μl of CCK-8 reagent was added to each well containing 100 μl MSCGM™ mesenchymal stem cell growth medium. hBM-MSCs were incubated with CCK-8 reagent for 2 h followed by reading with micro plate reader FLUOstar Omega (BMG Labtech) at the absorbance at 450 nm. The measurements were performed daily over a 6-day period. Each experiment was repeated 3 times with 3 repeats of each variant in a single experiment.

### Immunocytochemistry

hBM-MSC seeded at a density 5 × 10^3^ cells on 24-well plates, stained with Molday ION Rhodamine B™ and CellTracker™ Green CMFDA, or transfected with mRNA eEGFP on 2nd and 7th day of culture were washed three times with PBS and fixed by 15-min incubation in 4% paraformaldehyde (PFA; MP BIOMEDICALS) in PBS and then again washed three times in PBS. Permeabilization was accomplished by 3-min incubation in 0.05% Triton X100 (Triton®X-100 Sigma Ultra, Sigma-Aldrich) in PBS. Non-specific binding was blocked using 3% of albumin from bovine serum (BSA; Sigma-Aldrich) for 90 min. The cells were washed with PBS, and they were incubated overnight at 4 °C with primary antibodies against CD90 (1:250, Sigma-Aldrich), CD44 (1:500, Santa Cruz), SSEA4 (1:100, Millipore), and CXCR4 (1:250, Santa Cruz). Then, cells were washed three times with PBS, and they were exposed for 90 min (RT in the dark) to goat anti-mouse IgG (H+L)-488 (Invitrogen) or -546 (Invitrogen) secondary antibodies. Additionally, cell nuclei were stained with 5 μM Hoechst 33258 (Sigma-Aldrich). The images were captured using fluorescent microscope Axiovert.A1 Zeiss (Carl Zeiss MicroImaging GmbH). Following acquisition, images were processed using the Zeiss Axiovert.A1 software package v. ZEN blue software (Carl Zeiss MicroImaging GmbH).

### Flow cytometry of cell phenotype identification

To identify a phenotype of hBM-MSC labeled with Molday ION Rhodamine B™ and CellTracker™ Green CMFDA or transfected with mRNA eEGFP, we performed a flow cytometry analysis to examine the presence of cell markers CD90, CD44, SSEA4, and CXCR4 (BD Biosciences, Franklin Lakes, NJ, USA) in 2- or 7-day-long cultures after the labeling procedure. hBM-MSCs were detached from the culture vessel by trypsinization, suspended in PBS and centrifuged twice at 1200 rpm. Then, cells (5 × 10^5^) were incubated in 100 μl of buffer (PBS with 1%FBS and with 2 μl of CD90-PE, 5 μl of CD44-PE, 5 μl of SSEA4-PE, and 2 μl of CXCR4-PE antibodies for 30 min in the dark). Next, the cells were washed twice with buffer and then 300 μl of PBS was added to the cells. Cell fluorescence was analyzed by flow cytometry in a BD Canto II instrument, and the data were analyzed using FACSDiva software (BD) with compensated parameters. Additionally, the relative size (FSC) and granularity (SSC) of cells were assessed. The viability of hBM-MSC was measured in the 7AAD test. Moreover, the percentage of Molday ION Rhodamine B™, CellTracker™ Green CMFDA, eEGFP, CD90, CD44, SSEA4, and CXCR4-positive cells was evaluated. The intensity of the fluorescence signal was measured and expressed as an average for 1 × 10^4^ cells. All experiments were done in triplicate using an isotype control.

### hBM-MSC adipogenic assay

The cells labeled by CellTracker™ Green CMFDA and Molday ION Rhodamine B™ or transfected with Stemgent® eEGFP hBM-MSC were plated into a 24-well (1.5 × 10^5^ cells per well) plates. The cells were cultured in StemPro® Adipogenesis Differentiation Kit (Gibco) at 37 °C, 5% CO_2_ for 15 days. The culture medium was replaced every other day. The cells were washed three times with PBS and fixed for 30 min with 4% PFA and then washed three times with PBS. The evaluation of morphology and fluorescence of cells was carried out using the Axiovert.A1 Zeiss fluorescence microscope in the Zen blue program. Each experiment was repeated 3 times with 3 repeats of each variant in a single experiment. For each repeat, at least 5 pictures were counted. The analysis was performed in ImageJ (free software shared by National Institutes of Health (NIH)—https://imagej.nih.gov/ij).

### hBM-MSC osteogenic assay

hBM-MSCs were seeded (2 × 10^5^cells/flask) and grown on a T-25 bottle to achieve 70% confluence and then labeled with a suitable stain (Molday ION Rhodamine B™, CellTracker™ Green CMFDA or Stemgent® eEGFP) according to the procedures described above. Labeled cells were stripped from the bottom of the culture vessel with trypsin, centrifuged (3 min 1000*g*), and counted. hBM-MSCs were then plated in the number of 1 × 10^3^ on 24-well in MSCGM™ mesenchymal stem cell growth medium. Four hours later, the culture medium was exchanged to StemPro Osteogenesis Differentiation Kit, where the cells were cultured for another 3 weeks at 37 °C, 5% CO_2_. The culture medium was replaced every third day. After 21 days, cells were rinsed with DPBS (Lonza) and fixed in 4% PFA for 30 min. Cells were then washed twice with distilled water and stained with 2% Alizarin Red Solution (Millipore) for 3 min. Excess of the dye was eliminated by three times distilled water rinsing. Evaluation of morphology and fluorescence of cells was performed using the Axiovert.A1 Zeiss fluorescence microscope in the Zen blue software.

### hBM-MSC chondrogenic assay

hBM-MSCs were grown on a T-75 bottle to achieve 70% confluence and then labeled with a suitable label (Molday ION Rhodamine B™, CellTracker™ Green CMFDA, or Stemgent® eEGFP) according to the procedures described above. Labeled cells were detached from the bottom of the culture vessel with trypsin (Gibco) and centrifuged (3 min 1000*g*). The supernatant was removed by decantation, and hBM-MSCs harvested as pellet were seeded as 5-μl drops on the bottom of the 24-well. Each well contained 3 drops. Prepared cells were placed at 37 °C, 5% CO_2_ at very high humidity of 80–90% for 4 h. After the incubation period, StemPro Chondrogenesis Differentiation Kit (Gibco) was added to each well. hBM-MSCs were grown for another 15 days at 37 °C, 5% CO_2_. The culture medium was replaced every third day. After 15 days of culture, in order to confirm the differentiation of hBM-MSC into chondrocytes, the cells were fixed by 30-min incubation in 4% PFA, washed with DPBS, and stained for 30 min with 1% Alcian blue solution (Sigma-Aldrich). Cells were washed twice with 0.1 N HCl (Chempur) and placed in distillate water. Evaluation of cell morphology and fluorescence was carried out using the Axiovert.A1 Zeiss fluorescence microscope in the Zen blue software.

### Quantitative real-time PCR analysis

Total RNA was extracted from cells detached by trypsin, which were cultured on 25-cm^2^ flasks (2 × 10^5^cells/flask) at the 2nd, 5th, and 7th day after hBM-MSC staining with Molday ION Rhodamine B™ and CellTracker™ Green CMFDA or Stemgent® eEGFP using Trizol reagent (Invitrogen). Then, 4 μg of each sample was used in reverse transcription reaction High Capacity RNA-to-cDNA Kit (Applied Biosystems), following the producer instructions. Real-time PCR analysis was performed in ABI Prism 7500 Sequence Detection System using 30 μg of cDNA. The used primers are listed in Table 1. The reaction was performed using SYBR Green PCR Master Mix (Applied Biosystems). The fluorescent signal from transcripts was normalized against GAPDH gene level. The threshold cycle values (ΔCt) were quantified as fold changes by the 2^-ΔΔCt^ method [26].

**Table 1:** List of primers used in the qRT-PCR reaction

Gene	Primers sequence Forward Reverse
GDNF (glial cell line-derived neurotrophic factor)	TTTAGGTACTGCAGCGGCTCTT TCACTCACCAGCCTTCTATTTCTG
GAPDH (glyceraldehyde 3-phosphate dehydrogenase)	CCACATCGCTCAGACACCAT TGACCAGGCGCCCAATA
IGF (insulin-like growth factors)	TGCTTCCGGAGCTGTGATCTA GCTGACTTGGCAGGCTTGAG
EGF (epidermal growth factor)	GCAGAGGGATACGCCCTAAGT CAAGAGTACAGCCATGATTCCAAA
NT3 (neurotrophin 3)	AACATCACGGCGGAAACGGTAC ACTCTCACTGTCACATACCGAGTACTCC
BDNF (brain-derived neurotrophic factor)	ATTACAATCAGATGGGCCACATG AGGGAGAAAGCAGAAACAAGACA
PSAP (prosaposin)	AACAGACCAGGTGTCCTTGG CCATGTTAAAGGGCTCGTGT
CNTF (ciliary neurotrophic factor)	TGTGCGTGCTTGCATGTG ACCCTGAAGTGGAAGGACGTT
SEM (semaphorin)	CCCTGGGGAACTTCTACCTC TCGAAGTCTTGTTCCCTGCT
HGF (hepatocyte growth factor)	GCCCTATTTCTCGTTGTGAAGGT CTGTATCTCAAACTAACCATCCATCCTATG

### Enzyme-linked immunosorbent assay (ELISA)

hBM-MSCs (Lonza) were cultured at 75-cm^2^ flasks for five passages and labeled with Molday ION Rhodamine B™ and CellTracker™ Green CMFDA or transfected with mRNA GFP. To test the release of EGF, GDNF, IGF-1, and HGF, the concentration (or amount) of these proteins was measured in media taken from native and labeled cells cultured in serum-free Opti-Mem® I (1x) Reduced Serum Medium (Life Technologies) for 24 h. At the 2nd, 5th, and 7th day after hBM-MSC staining, the medium was collected and thickened by centrifugation (30 min, 3000×*g*, RT) in Vivaspin® concentrators with PES 5000 or 10,000 MWCO membranes (Sartorius). Then, the protein content was analyzed using the Bradford method with absorbance level read on micro plate reader FLUOstar Omega (BMG Labtech) at 450 nm. The assessment of human EGF, GDNF, IGF-1, and HGF secretion into serum-free medium was performed using specific ELISA kits: human EGF ELISA Kit, human GDNF ELISA Kit; human IGF1 ELISA Kit, and human HGF ELISA Kit (Abcam) following the manufacturer’s instructions with 100 μg of total protein placed on each well of 96-well ELISA plate.

### Statistical analysis

Results of qRT-PCR (quantitative real-time polymerase chain reaction) analysis are expressed as the mean and standard deviation (S.D.). In order to evaluate the statistical significance of differences between mean values, analysis of variance (ANOVA) and Bonferroni’s multiple comparisons test were performed. GraphPad Prism software was used for all calculations. All the remaining statistical calculations were performed using PROC MIXED (SAS 9.4). The type III test of fixed effects has been used to determine statistical significance, and the least mean square (LMS) difference test was employed for comparison between means. The hierarchical statistical models were set to fit the data structure (the replications of experiments were treated as random variable), while graphs present pooled results of all replications. In all experiments, the level of statistical significance has been set at *p* < 0.05.

## Results

### Characterization of hBM-MSC

**Fig. 1:**
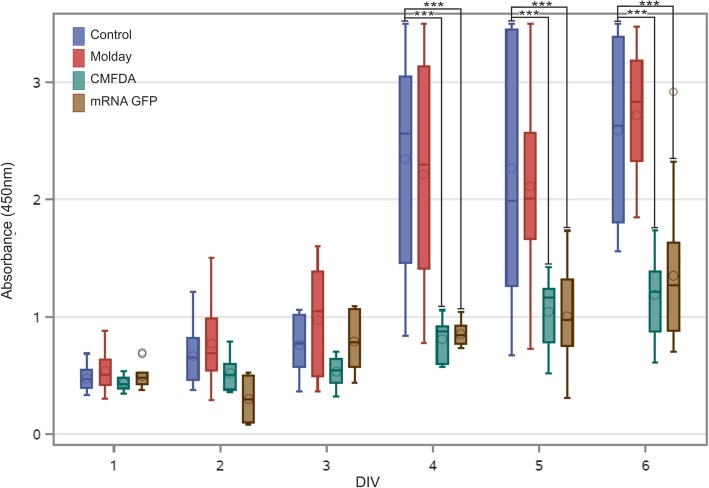
The metabolic activity level of hBM-MSC labeled with various vital stains. The upper and lower bounds of the box are the first and third quartiles, with the median drawn as a line inside the box. Its whiskers extend from the box to the fences, which are placed at ± 1.5 interquartile range units. Data points beyond the fences are considered outliers and are shown as circles. The circles inside the boxes point on means. *p < 0.05, **p < 0.01, ***p < 0.001 (*n* = 5)

**Fig. 2:**
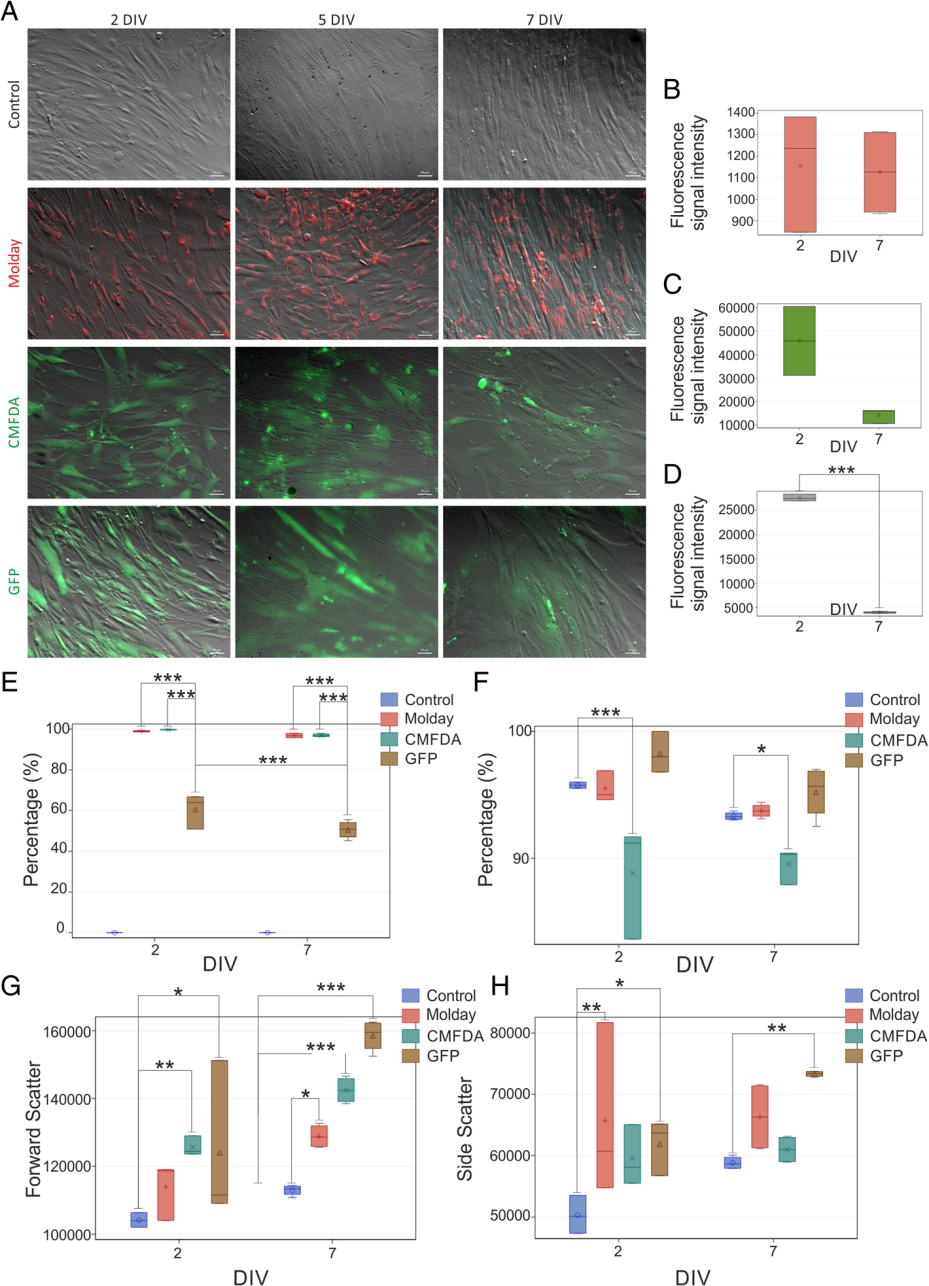
Microscopic analysis of labeled hBM-MSC. **a** Vital observation of hBM-MSC stained with Molday ION Rhodamine B™ (Molday), CellTracker™ Green CMFDA (CMFDA), and eGFP (mRNA GFP) over 7 days in vitro culture. *DIV, day in vitro*.* Scale 50 μm. Measurement of fluorescence signal intensity generated by cells stained with **b** Molday ION Rhodamine B™ (Molday), **c** CellTracker™ Green CMFDA (CMFDA), and **d** transfected with mRNA eGFP on the second and seventh day of in vitro culture. **e** Comparison of percentage of fluorescent hBM-MSC in all groups. The viability of cells assessed in 7AAD test on the second and seventh day after labeling (**f**). The measurement of relative size (**g**) and granularity (H) of cells. *p < 0.05, **p < 0.01, ***p < 0.001 (*n* = 3–4)

The unlabeled hBM-MSC had a specific pattern of metabolic activity with a slow increase over the first 3 days followed by a jump on day 4 and a further slow increase until the end of the experiment at day 6 (Fig. 1). hBM-MSC labeled with Molday ION Rhodamine B™ featured exactly the same characteristic of metabolic activity, with no statistical difference at any time point. While the hBM-MSC labeled with CellTracker™ Green CMFDA and transfected with eGFP-mRNA matched the level of metabolic activity of unlabeled hBM-MSC over the period of slow growth during the first 3 days, they did not show up the jump of metabolic activity on day 4 and their metabolic activity was slowly increasing until day 6. Therefore, the metabolic activity of hBM-MSC labeled with CellTracker™ Green CMFDA and transfected with eGFP-mRNA was statistically lower from day 4 until the end of the experiment, and no difference was observed in this parameter between CellTracker™ Green CMFDA and eGFP-mRNA during the entire period of the experiment. These results may indicate cytotoxic effect of CellTracker™ Green CMFDA and probably mRNA eGFP labeling on hBM-MSC. This result may be associated with reduction of cell metabolism as well as decreased viability or proliferation rate of hBM-MSC observed after staining. Vital observation of hBM-MSC stained by CellTracker™ Green CMFDA revealed morphological changes accompanied the decreased metabolic activity. We observed that during the 7-day culture, some of the cells lose typical for MSC, spindle-like shape, shrink, become round and detach from the bottom of the culture plate. eGFP-transfected and Molday ION Rhodamine B™-stained cells maintain correct shape, consistent with ones observed in control non-labeled hBM-MSC **(**Fig. 2a). hBM-MSC labeled with Molday ION Rhodamine B™ preserved the intensity of the fluorescent signal for 7 days after staining. However, in terms of hBM-MSC labeled with CellTracker™ Green CMFDA or transfected with eGFP, the decrease in fluorescence signal intensity, respectively 69% and 86%, was noticed after 7 days of cell labeling (Fig. 2b–d). Flow cytometry analysis revealed that almost all hBM-MSC labeled with Molday ION Rhodamine B™ (99.0 ± 0.29% after 2 days and 97.0 ± 0.66% after 7 days after labeling) presented the fluorescence; however, among hBM-MSC population transfected with eGFP, the percentage of positive cells was much lower (respectively 60.0 ± 6.93% after 2 days and 51.0 ± 3.83% after 7 days of cell transfection) (Fig. 2e). The viability of hBM-MSC labeled with CellTracker™ Green CMFDA was significantly lower after 2 or 7 days in culture (respectively 88.0 ± 3.71% and 89.5 ± 1.16%) than among control hBM-MSC or cells labeled with Molday ION Rhodamine B™ or transfected with eGFP (respectively 95.75 ± 0.2%, 95.5 ± 1.0%, and 98.27 ± 1.81) after 2 days and (93.3 ± 0.27%, 93.73 ± 0.48%, and 95.2 ± 1.81 after 7 days in culture) (Fig. 2f). To further identify the influence of labeling on hBM-MSC, cultured cells stained with Molday ION Rhodamine B™ and CellTracker™ Green CMFDA or transfected with eGFP were tested for their size and granularity. The relative size of hBM-MSC labeled with all three stains was much bigger after 7 days of observation in comparison to the control non-labeled cells, and the FSC values were respectively 128,856.5 ± 3085.65 for Molday ION Rhodamine B™-labeled cells, 142,477.0 ± 3431.88 for hBM-MSC labeled with CellTracker™ Green CMFDA, 158,542.25 ± 4099.13 for eGFP-transfected cells, and 112,897.5 ± 1372.43 for control, non-labeled hBM-MSC (Fig. 2g). This corresponded to the increase of SCC values describing granularity of Molday ION Rhodamine B™ and eGFP-labeled hBM-MSC compared to non-labeled cells (respectively 66,315.0 ± 5039.17; 73,329.25 ± 405.07; and 58,814.75 ± 925.89) detected after 7 days in vitro (Fig. 2h).

### The basic immune phenotype of labeled hBM-MSC

**Fig. 3:**
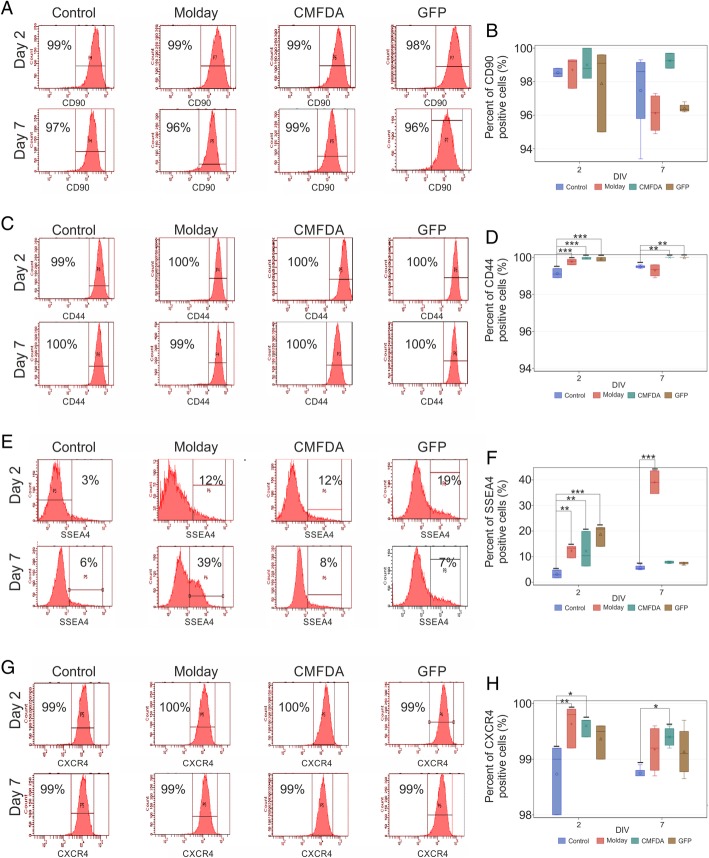
Phenotypical analysis of hBM-MSC in the 2nd and 7th day of culture after staining with CellTracker™ Green CMFDA (CMFDA), eGFP (mRNA GFP), and Molday ION Rhodamine B™ (Molday) with antibodies directed against proteins: CD90, CD44, SSEA4, and CXCR4 performed by cytometric analysis (**a**, **c**, **e**, **g**). The bar charts (**b**, **d**, **f**, **h**) show the comparison of the percentage of hBM-MSC positive for particular antigen among control, CellTracker™ Green CMFDA (CMFDA), eGFP (mRNA GFP), and Molday ION Rhodamine B™ (Molday)-stained cell groups. *p < 0.05, **p < 0.01, ***p < 0.001 (*n* = 3–4)

Immunocytochemical analysis of hBM-MSC cells labeled with Molday ION Rhodamine B™ and CellTracker™ Green CMFDA or transfected with eGFP showed that labeled cells maintained their basic immune phenotype with only minor disturbances (Additional file 1: Figure S1). The expression of CD90 and CD44 was consisted with characteristic surface markers of control hBM-MSC. Almost all labeled hBM-MSC expressed both antigens on their surface 2 and 7 days after cell staining (Fig. 3a–d). We noticed the elevation of SSEA4 expression in hBM-MSC labeled with Molday ION Rhodamine B™ and CellTracker™ Green CMFDA or transfected with mRNA eGFP in comparison to non-labeled cells (respectively 12 ± 1.98%, 12 ± 5.7%, 19.0 ± 3.36%, and 3.0 ± 3.36%) after 2 days of staining. Interestingly, further growth of the percentage of SSEA4-positive cells labeled with Molday ION Rhodamine B™ was observed after 7 days of cell labeling (39.0 ± 4.48%) (Fig. 3e, f). In terms of CXCR4, flow cytometry analysis revealed that all hBM-MSC labeled with Molday ION Rhodamine B™ and CellTracker™ Green CMFDA or transfected with mRNA eGFP retained high expression of this protein observed in control cells in both time points of culture (Fig. 3g, h).

### The influence of hBM-MSC labeling on adipogenesis

**Fig. 4:**
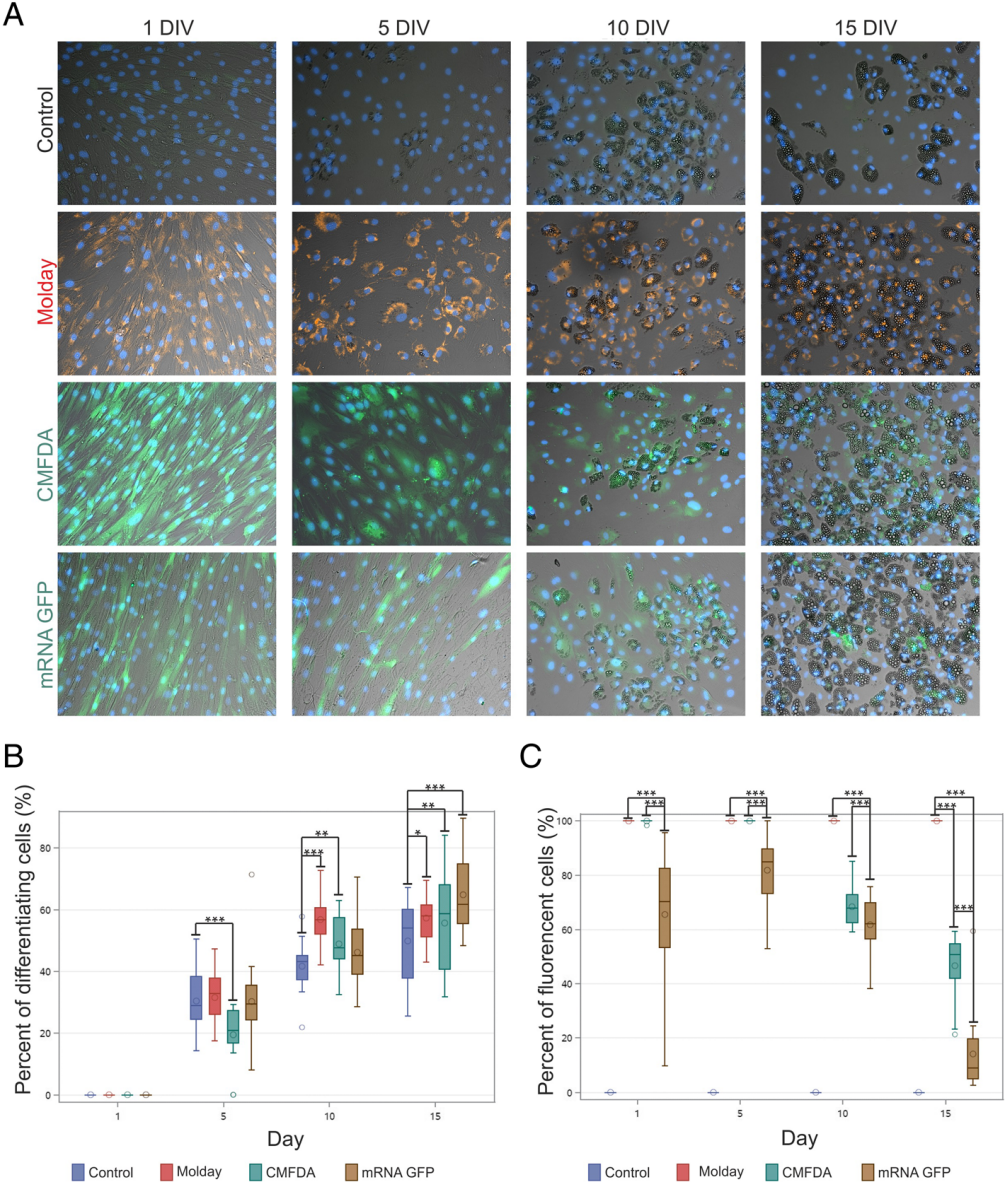
Adipogenesis of hBM-MSC labeled with Molday ION Rhodamine B™ (Molday), CellTracker™ Green CMFDA (CMFDA), and eGFP (mRNA GFP). Adipogenesis occurred in all cell populations (**a**); however, the percentage of differentiating cells (**b**) and persistence of particular dyes (**c**) were significantly different among cell group. *p < 0.05, **p < 0.01, ***p < 0.001 (*n* = 3)

During the process of adipogenesis of non-stained (control), Molday ION Rhodamine B™-labeled, CellTracker™ Green CMFDA-labeled, or mRNA eGFP-transfected hBM-MSC, cells underwent changes in morphology including the formation of fat drops within their cytoplasm, which were present from the fifth day of culture (Fig. 4a). In case of all types of hBM-MSC, the percentage of differentiating cells significantly grew from day 5 to 15, except of stabilization observed in Molday ION Rhodamine B™-labeled group between 10th and 15th day. All cell groups were able to complete the differentiation process. On the 5th day of adipogenesis, the group of CellTracker™ Green CMFDA-labeled cells contained smaller percent of differentiating cell population than the control, Molday ION Rhodamine B™-labeled, and mRNA eGFP-transfected hBM-MSC. However, on the 10th day, both Molday ION Rhodamine B™-labeled and CellTracker™ Green CMFDA-labeled cells consisted of a higher proportion of adipocytes in comparison to the control cells, whereas mRNA eGFP differentiating cell percent was comparable to the control level. Simultaneously, CellTracker™ Green CMFDA-labeled and mRNA eGFP-transfected cells had a lower percent of differentiating cells than Molday ION Rhodamine B™-labeled hBM-MSC. The elevated percentage of differentiating cells in all three stained hBM-MSC groups in comparison to the control non-labeled hBM-MSC was observed on day 15 of adipogenesis. Surprisingly, the mRNA eGFP-transfected group on day 15 revealed the highest percentage of differentiating cells (Fig. 4b).

During adipogenesis of the CellTracker™ Green CMFDA-labeled and mRNA eGFP-transfected hBM-MSC, there was a decrease in the percent of fluorescent cells. On day 1 of differentiation, the percentage of eGFP-positive cells was lower than that of Molday ION Rhodamine B™-positive and CellTracker™ Green CMFDA-positive hBM-MSC, which both labeled 100% of cells. On day 5, there was a significant increase in the proportion of fluorescent cells in the mRNA eGFP-transfected cell group which together with the decrease of general cell number (data available in Additional file 2: Figure S2) indicates on the extermination of non-fluorescent cells from the group of eGFP-labeled hBM-MSC. However, the percentage of fluorescent cells remained still lower than that observed in Molday ION Rhodamine B™- and CellTracker™ Green CMFDA-labeled hBM-MSC. On the 10th day of adipogenesis, we observed a drop of the percentage of fluorescent cells in both CellTracker™ Green CMFDA- and eGFP-labeled cells. mRNA eGFP-transfected cells were characterized by the lowest percentage of fluorescent cells in comparison to CellTracker™ Green CMFDA- and Molday ION Rhodamine B™-labeled cells. This proportion was maintained on day 15. In the case of hBM-MSC transfected with mRNA eGFP, the signal from eGFP protein almost completely disappeared after 15 days of the experiment. About 50% of CellTracker™ Green CMFDA-labeled cells remained fluorescent during adipogenesis, whereas the number of differentiating cells labeled with Molday ION Rhodamine B™ remained comparable to their original amount and equaled impressive 100% of cells after 15 days of differentiation (Fig. 4c). During the differentiation, the cells do not proliferate, so mitosis-related label dilution was not taken into consideration.

### The influence of vital staining on osteo- and chondrogenesis of hBM-MSC

**Fig. 5:**
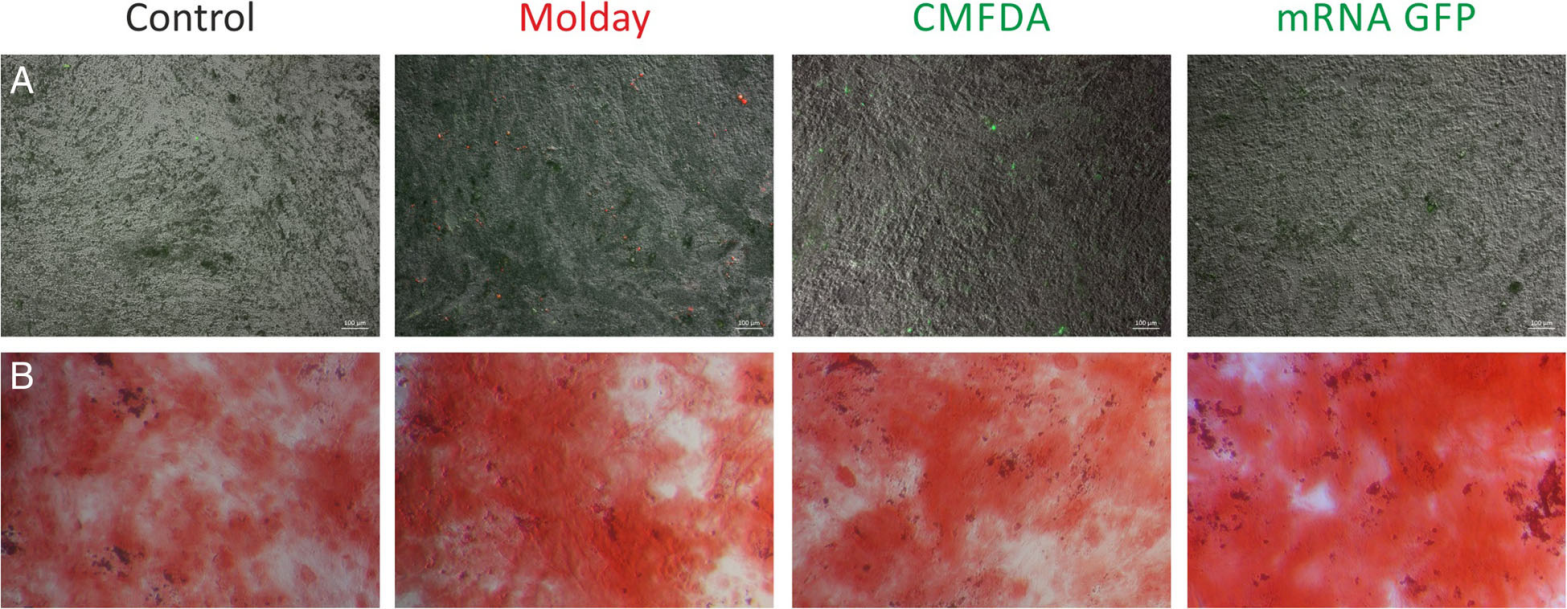
Osteogenesis of control, Molday ION Rhodamine B™ (Molday)-labeled, CellTracker™ Green CMFDA (CMFDA)-labeled, and mRNA eGFP (mRNA GFP)-transfected hBM-MSC. All cells were able to undergo osteogenic differentiation (**b**); however, none of them maintained fluorescence (**a**)

**Fig. 6:**
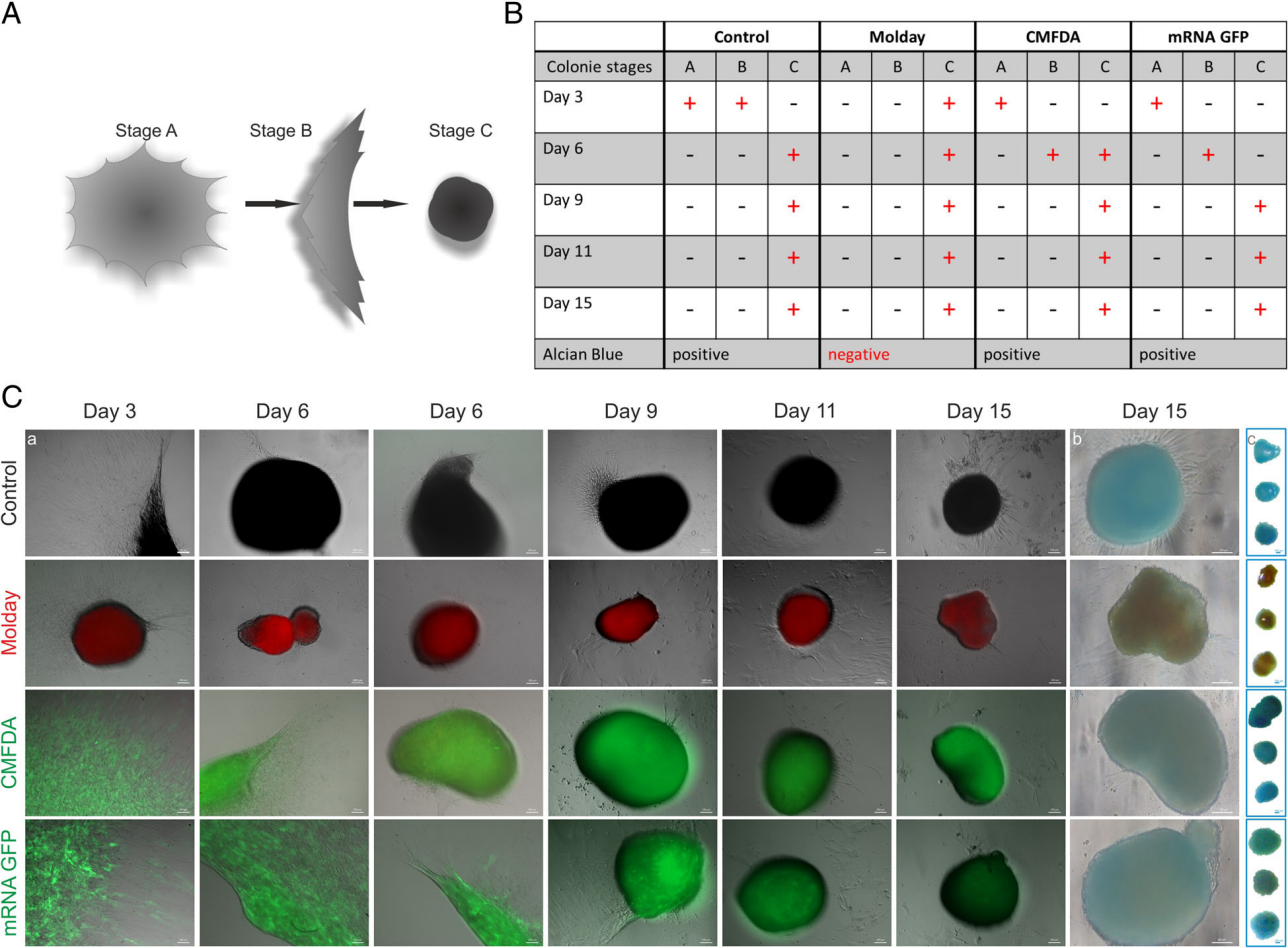
Chondrogenesis of control, Molday ION Rhodamine B™ (Molday)-labeled, CellTracker™ Green CMFDA (CMFDA)-labeled, and mRNA eGFP (mRNA GFP)-transfected hBM-MSC. **a** A schema shows the developmental stages of micromass creation observed during hBM-MSC osteogenic differentiation. **b** Table contains data about the presence of particular colony stages during differentiation period in stained hBM-MSC groups. **c** Pictures of hBM-MSC colonies visible in the fluorescent and light microscope with Alcian blue staining on day 15

All three tagged hBM-MSC populations were able to complete the osteogenic differentiation, which was manifested by alkaline phosphatase synthesis after 21 days of cell culture in the osteogenetic stimulating medium, but the fluorescent signal from all three markers disappeared during differentiation (Fig. 5). Under the influence of chondrogenesis-stimulating medium in all experimental variants, cells have formed typical for chondrogenesis micromasses, but the dynamics of this process was different for hBM-MSC stained with individual dyes. Molday ION Rhodamine B™-labeled cells were faster in creation of a fully formed micromasses than control cells, but in the case of CellTracker™ Green CMFDA-tagged cells and mRNA eGFP-transfected hBM-MSC, the time period necessary for this process was elongated. Three stages of micromass formation were observed. Stage A consisted of cells growing in the form of flat colonies with spherical shape and very high density of cells in the central part of the colonies. During stage B, colonies were shrinking and folding one of the sides forming a colony of morphology close to triangular. In the C stage, colonies formed a three-dimensional sphere-like structure (Fig. 6a). Within the first 3 days of differentiation process, only Molday ION Rhodamine B™-labeled cells reached stage C, while control cells’ colonies were at stages A and B. The CellTracker™ Green CMFDA -labeled and mRNA eGFP-transfected cells have reached only stage A. On day 6, the control and the Molday ION Rhodamine B™-labeled cells’ colonies were in stage C while mRNA eGFP-transfected colonies reached stage B. Colonies of CellTracker™ Green CMFDA-labeled cells were in stage B or C. On day 9 of differentiation, the colonies in all variants of the experiment reached stage C and this state has sustained until the end of the experiment on day 15 (Fig. 6b). The fluorescence of labeled cells was maintained for all used tags. Staining of the colonies using Alcian blue showed that control hBM-MSC and cells labeled with CellTracker™ Green CMFDA and transfected with mRNA eGFP synthesized proteoglycans indicating their differentiation into chondrocytes, whereas the Molday ION Rhodamine B™-labeled hBM-MSC did not reveal blue color. It could be related to the imaging interference due to the presence of dark iron oxide nanoparticles, or less probable these cells did not undergo the full chondrogenic differentiation (Fig. 6c).

### Analysis of the level of transcript encoding growth factors secreted by hBM-MSC after labeling with the various vital dyes

**Fig. 7:**
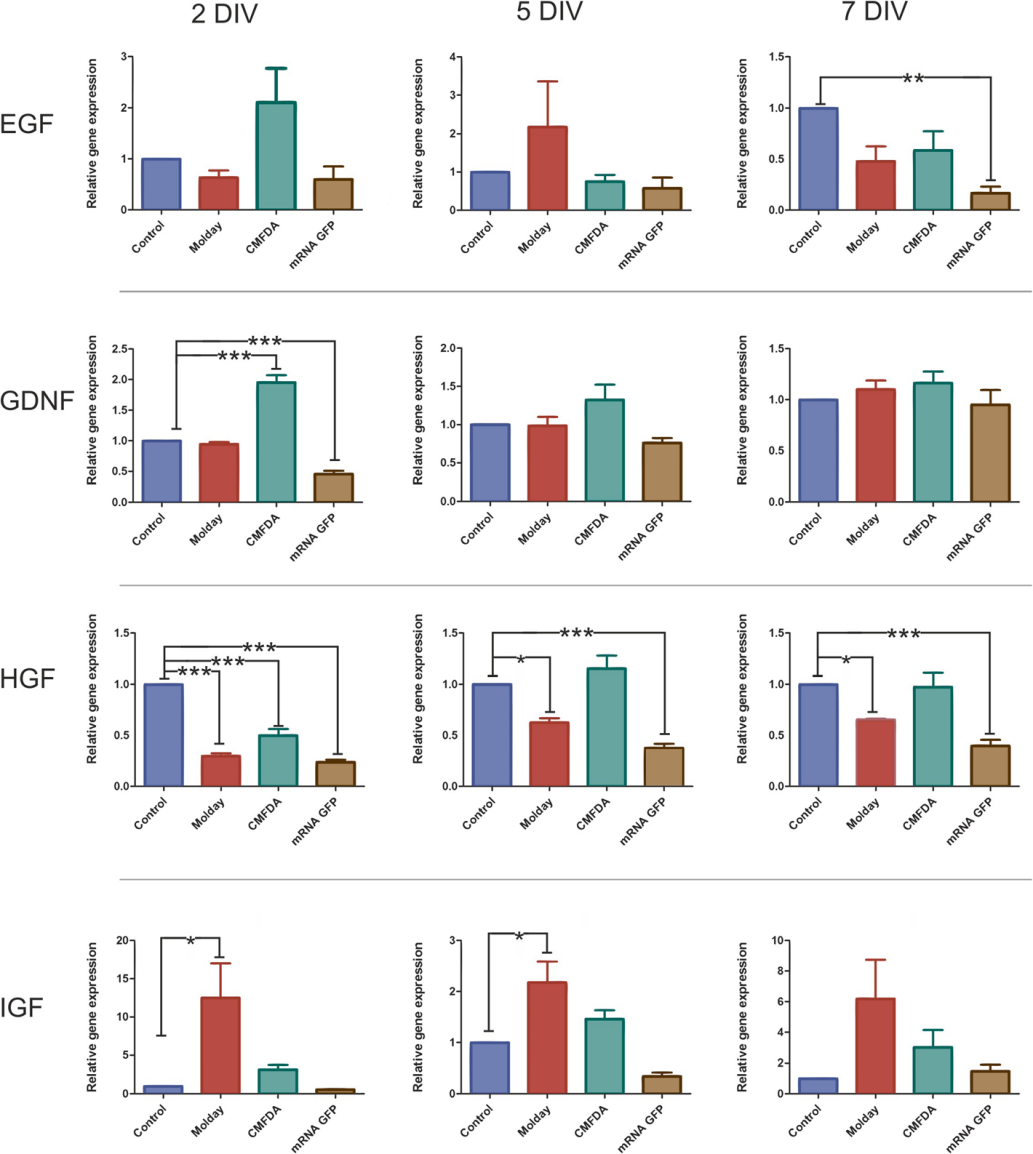
The real-time PCR analysis of growth factors’ transcript level in cells stained with Molday ION Rhodamine B™ (Molday), CellTracker™ Green CMFDA (CMFDA), and mRNA eGFP (mRNA GFP) in comparison to unlabeled hBM-MSC in the 2nd, 5th, and 7th day after labeling. *p < 0.05, **p < 0.01, ***p < 0.001 (*n* = 5–7)

Quantitative real-time PCR (qRT-PCR) analysis of transcript-level coding growth factors such as *BDNF*, *CNTF*, *EGF*, *GDNF*, *HGF*, *IGF*, *NT3*, *PSAP*, and *SEM* was performed for the control and CellTracker™ Green CMFDA-, mRNA EGFP-, or Molday ION Rhodamine B™-labeled hBM-MSC. The material was collected at several time points—after 2, 5, and 7 days of culture. The relative expression level of most of the investigated growth factors remained stable after cell labeling (*BDNF*, *CNTF*, *NT3*, *PSAP*, *SEM*—data available in Additional file 3: Figure S3); however, we have observed the significant changes in the case of *EGF*, *GDNF*, *HGF*, and *IGF* gene expression (Fig. 7). The most profound alterations occurred on the second day of culture. The expression level of GDNF was increased in the case of CellTracker™ Green CMFDA-labeled cells and decreased in mRNA eGFP-transfected hBM-MSC. These changes were temporary and absent on 5th and 7th day of culture. Moreover, on the second day, the IGF expression level in Molday ION Rhodamine B™-labeled cells was highly elevated. This state maintained from the second to the 5th day of cell culture; however, on the 7th day, the results become statistically insignificant due to high variability. On the second day, all labeled cells had decreased expression level of *HGF* gene. *HGF* gene expression in CellTracker™ Green CMFDA-labeled hBM-MSC aligned with the control cell level on day 5, while in the case of Molday ION Rhodamine B™-labeled and mRNA eGFP-transfected hBM-MSC, it remained decreased to 7th day. Moreover, the *EGF* expression level, which was lower in the case of mRNA eGFP-transfected cells from the second day, became significantly decreased on the 7th day of culture in this cell group. In summary, most of gene expression level alterations vanished with time; however, in the 7th day of culture, mRNA level for HGF was still affected in Molday ION Rhodamine B™-labeled cells while *EGF* and *HGF* transcript level was decreased in eGFP-transfected hBM-MSC.

### Changes in growth factor release by hBM-MSC after labeling with selected vital dyes

**Fig. 8:**
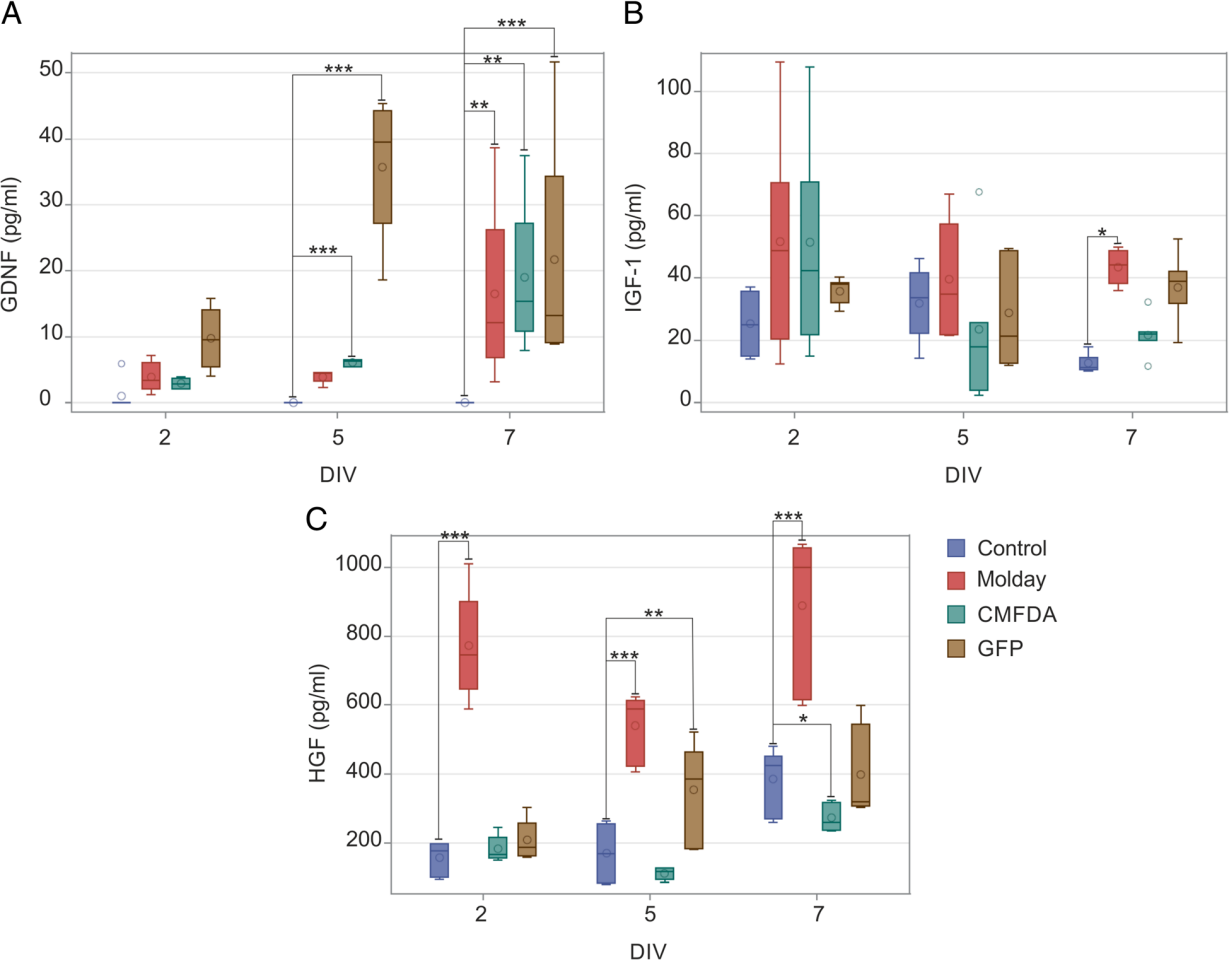
ELISA analysis of GDNF (**a**), IGF-1 (**b**), and HGF (**c**) level in culture media obtained from hBM-MSC stained with Molday ION Rhodamine B™ (Molday), CellTracker™ Green CMFDA (CMFDA), and mRNA eGFP (mRNA GFP) collected on the 2nd, 5th, and 7th day after labeling as compared to unlabeled hBM-MSC. *p < 0.05, **p < 0.01, ***p < 0.001 (*n* = 6)

Due to the positive results obtained in qRT-PCR analysis, we have examined protein release of these growth factors in media of cultured hBM-MSCs after different cell staining. ELISA performed in native (control), Molday ION Rhodamine B™-labeled, and CellTracker™ Green CMFDA-labeled, or mRNA GFP-transfected hBM-MSC culture medium on the 2nd, 5th, and 7th day after staining has shown that the level of GDNF was not different in any cell group on the second day; however, it was significantly higher in CellTracker™ Green CMFDA-labeled or mRNA GFP-transfected hBM-MSC culture medium on the 5th day. At the 7th day, the increase of GDNF amount was observed in all labeled cell groups as compared to the native hBM-MSC (Fig. 8a). In terms of IGF-1, the level of this protein was elevated on the 7th day after labeling in culture media collected from Molday ION Rhodamine B™-stained hBM-MSC, whereas the amount of IGF-1 remained close to the control value in other two groups (Fig. 8b). The most profound effect on HGF release was observed in the case of Molday ION Rhodamine B™-stained hBM-MSC, with significant secretion elevation in all investigated time points. Moreover, on the 5th day after transfection, the increased HGF level was observed in GFP-labeled cells, whereas it was decreased in CellTracker™ Green CMFDA-marked cells on the 7th day after labeling (Fig. 8c). However, the EGF level determined in media obtained from all variants of hBM-MSC staining was below the detection threshold (data not shown).

## Discussion

We have shown that labeling of hBM-MSC with different stains might affect their characteristics. The results of our studies reveal that Molday ION Rhodamine B™ seems to be a preferred stain for labeling of hBM-MSC. In contrast to eGFP-mRNA and CMFDA-labeled cells, Molday ION Rhodamine B™ does not affect the metabolic activity of hBM-MSC in standard culture conditions. Interestingly, we have observed the increase of SSEA4 antigen (characteristic for cells in the early developmental stage, i.e., embryonic cells) expression in hBM-MSC labeled with Molday ION Rhodamine B™. The data reported by other authors have shown that hBM-MSC cultured on gelatine-coated plates in serum-free medium supplemented by bFGF demonstrated high expression of SSEA4 accompanying augmented proliferation [27]. Increased proliferation after the introduction of iron nanoparticles into cells was in turn observed in neural progenitors cultured in the form of neurospheres [28]. The enhanced expression of SSEA4 observed in hBM-MSC after Molday ION Rhodamine B™ labeling presented in our studies may offset the potentially negative consequences of the process of cell labeling and by this way avoiding the drop of metabolic activity observed due to other stains investigated by us, but verification of this hypothesis would require additional studies, including single-cell analysis of cells, which acquired SSEA4 expression.

To further identify the influence of cell labeling, hBM-MSCs stained with Molday ION Rhodamine B™ and CellTracker™ Green CMFDA or transfected with eGFP were tested for the multipotency. Cells were induced in vitro using the adipogenic, osteogenic, and chondrogenic media. The data presented here demonstrated enhanced adipogenesis of hBM-MSC labeled with all three vital stains as compared to control cells, measured after 15 days of the assay. However, the Molday ION Rhodamine B™ was the only label capable permanent tagging of hBM-MSC throughout the entire process of adipogenic differentiation. In terms of osteogenic potential of hBM-MSC stained with Molday ION Rhodamine B™ and CellTracker™ Green CMFDA or transfected with eGFP, we have not observed any changes in osteogenic differentiation due to labeling procedure. All population of hBM-MSC tagged with different labels were stained positive to Alazirin red and showed calcium formation of an osteocyte phenotype after 3 weeks of osteogenic stimulation. There was also a positive chondrogenic process of hBM-MSC labeled with Molday ION Rhodamine B™ and CellTracker™ Green CMFDA or transfected with eGFP, suggesting that these cells could differentiate into chondrogenic cells similarly to controlled non-labeled cells. However, the reaction with Alcian blue revealed different chondrogenic formation among cell populations labeled with different stains. Molday ION Rhodamine B™-labeled hBM-MSC accelerated to day 3 achieving stage C of chondrogenesis, which was observed at day 6 in control cells, and slowed to day 9 in hBM-MSC stained with CellTracker™ Green CMFDA or transfected with eGFP. The lack of blue color after Alcian staining is probably due to the interference with dark color of iron oxide nanoparticles, but more advanced methods including an array of chondrogenesis-specific transcription factors would be warranted to bring final conclusions, especially as it was previously reported that other iron oxide nanoparticles (Feridex) selectively inhibited or altered the process of chondrogenesis in vitro [19, 29]. The other authors reported only slight alterations but in both osteogenesis and chondrogenesis, but not in adipogenesis in MSC labeled with Feridex [30]. Another type of SPIO—ferucarbotran—revealed a significant impact on osteogenic differentiation while adipogenesis and chondrogenesis were not investigated [31].

Molday ION Rhodamine B™, CellTracker™ Green CMFDA, or eGFP used in our studies to stain hBM-MSC did not significantly affect the expression of several growth factors. This was also noticed by other authors showing that SPIO labeling of MSC was neutral to different growth factor expression [32, 33]. Interestingly, we observed the reduction of mRNA level coding *HGF* expressed by hBM-MSC after staining with all three labels. It was in accordance with the previous findings of Bashar et al. who detected a lower level of *HGF* expression in MSC labeled with SPIO [34]. Surprisingly, we noticed an elevated level of *HGF* protein released by labeled hBM-MSC, most visible after Molday ION Rhodamine tracing. These results suggest the existence of unknown variables, such as both, iron oxide core as well as coating, and potentially method of cell culture and labeling, which may affect the growth factor expression and release. Therefore, further investigations on this topic are warranted, and until the reason is found, we suggest performing growth factor production evaluation at each experimental setting prior to proceeding with transplantation of iron oxide-labeled cells. Nevertheless, due to its ferromagnetic characteristics, SPIO enables cells to be visualized also in vivo using MRI even in deep locations both in animal models and in clinical scenario [35].

We have also observed the increase in size of labeled cells. This is particularly important for intra-arterial delivery as cell size determines the safety of the procedure [36].

While the selection of Molday ION Rhodamine B™ as the hBM-MSC label is indisputable, at the same time, we would like to warn the users of CellTracker™ Green CMFDA, which is routinely used in many studies, about potential negative consequences which were beyond what we expected [37, 38]. Other investigators have also reported similar problems with the use of lipophilic fluorescent dyes. Most of them require suspension in DMSO before use which can cause cytotoxic stress [39]. Moreover, the fast decrease of fluorescent signal and alteration of proliferation pattern are commonly known defects connected with fluorescent dyes. The disappearance of the signal during chondrogenic MSC differentiation was also observed [40]. We suggest performing similar to ours extensive analysis prior to using any cell label in experiments, as it can thoroughly bias results. The concept of using eGFP-mRNA as a cell label was rather new, but it did not live up to our expectations, especially in the efficacy and durability of labeling. While the process of labeling is relatively simple, the cost is rather high. In most articles, the expression of GFP protein in MSC was induced by the introduction of GFP gene via viral transfection. This was connected with impairment of cell proliferation ascribed to virus-related toxicity; however, in our study, we observed similar effect using the non-viral method [41]. Interestingly, while eGFP-mRNA transfection in many ways affected hBM-MSC similarly to CellTracker™ Green CMFDA, in some readout-like adipogenesis, the transfection resulted with fewer alterations than membrane labeling. The particular advantage of eGFP-mRNA would be if it is required to report on cell survival over a few days (no translation is present in dead cells, and half-life of eGFP is relatively short).

## Conclusions

Our study showed that basic hBM-MSC characteristic and functions might be affected by labeling. We observed strong alterations of metabolic activity and morphology after eGFP and Cell Tracker™ Green CMFDA hBM-MSC staining. Molday ION Rhodamine B™ labeling revealed superior properties relatively to other vital stains. The cell surface markers, proliferation, and multipotency of hBM-MSC labeled with Molday ION Rhodamine B™ are consistent with the characteristics of control hBM-MSC. Also, the relative expression level of most of the investigated growth factors remained stable after Molday ION Rhodamine B™ cell labeling. Based on the results of our study, we strongly recommend taking caution and carefully match the type of hBM-MSC tag for particular applications with consideration of their influence on cell properties.

## Additional files

Additional file 1: **Figure S1.** Phenotypical analysis of hBM-MSC in the 2nd and 7th day of culture after staining with CellTracker™ Green CMFDA (CMFDA), eGFP (mRNA GFP), and Molday ION Rhodamine B™ (Molday) with antibodies directed against proteins: CD90, CD44, SSEA4, and CXCR4 performed by immunocytochemistry. Scale 50 μm. (PNG 5939 kb)

Additional file 2: **Figure S2.** The number of cells in particular groups during adipogenesis of hBM-MSC labeled with Molday ION Rhodamine B™ (Molday), CellTracker™ Green CMFDA (CMFDA), and eGFP (mRNA GFP). (JPG 178 kb)

Additional file 3: **Figure S3.** Real-time PCR analysis of growth factors’ transcript level in cells stained with Molday ION Rhodamine B™ (Molday), CellTracker™ Green CMFDA (CMFDA), and mRNA eGFP (mRNA GFP) in comparison to unlabeled hBM-MSC in the 2nd, 5th, and 7th day after labeling, in which no statistically significant changes were observed. *p < 0.05, **p < 0.01, ***p < 0.001 (*n* = 5–7). (JPG 564 kb)

## Data Availability

Data sharing is not applicable to this article as no datasets were generated or analyzed during the current study. The datasets used and/or analyzed during the current study are available from the corresponding author on reasonable request.

## Abbreviations

ANOVA: Analysis of variance
BDNF: Brain-derived neurotrophic factor
bFGF: Basic fibroblast growth factor
CCK-8: Cell proliferation and cytotoxicity assay
CD: Cluster of differentiation
cDNA: Complementary DNA
CMFDA: 5-Chloromethylfluorescein diacetate
CNTF: Ciliary neurotrophic factor
CXCR4: C-X-C chemokine receptor type 4
DIV: Day in vitro
DMSO: Dimethyl sulfoxide
DNA: Deoxyribonucleic acid
DPBS: Dulbecco’s phosphate-buffered saline
EGF: Epidermal growth factor
eGFP: Enhanced GFP
FBS: Fetal bovine serum
FSC: Forward scatter
GAPDH: Glyceraldehyde 3-phosphate dehydrogenase
GDNF: Glial cell line-derived neurotrophic factor
GFP: Green fluorescence protein
hBM-MSCs: Human bone marrow mesenchymal stem cells
HGF: Hepatocyte growth factor
IGF: Insulin-like growth factors
ION: Iron oxide nanoparticles
LSM: Last mean square
MCGS: Mesenchymal cell growth supplement
MRI: Magnetic resonance imaging
mRNA: Messenger ribonucleic acid
MSCs: Mesenchymal stem cells
NT3: Neurotrophin 3
PBS: Phosphate buffer
PFA: Paraformaldehyde
PSAP: Prosaposin
qRT-PCR: Quantitative real-time polymerase chain reaction
S.D.: Standard deviation
SEM: Semaphorin
SPIO: Superparamagnetic iron oxide nanoparticles
SSC: Side scatter
SSEA4: Stage-specific embryonic antigen 4
