# Supplementary figures and images for: Labeling of human mesenchymal stem cells with different classes of vital stains: robustness and toxicity
Source: Stem Cell Res Ther. 2019 Jun 25;10:187. doi: 10.1186/s13287-019-1296-8 (PMC6593614; doi:10.1186/s13287-019-1296-8)

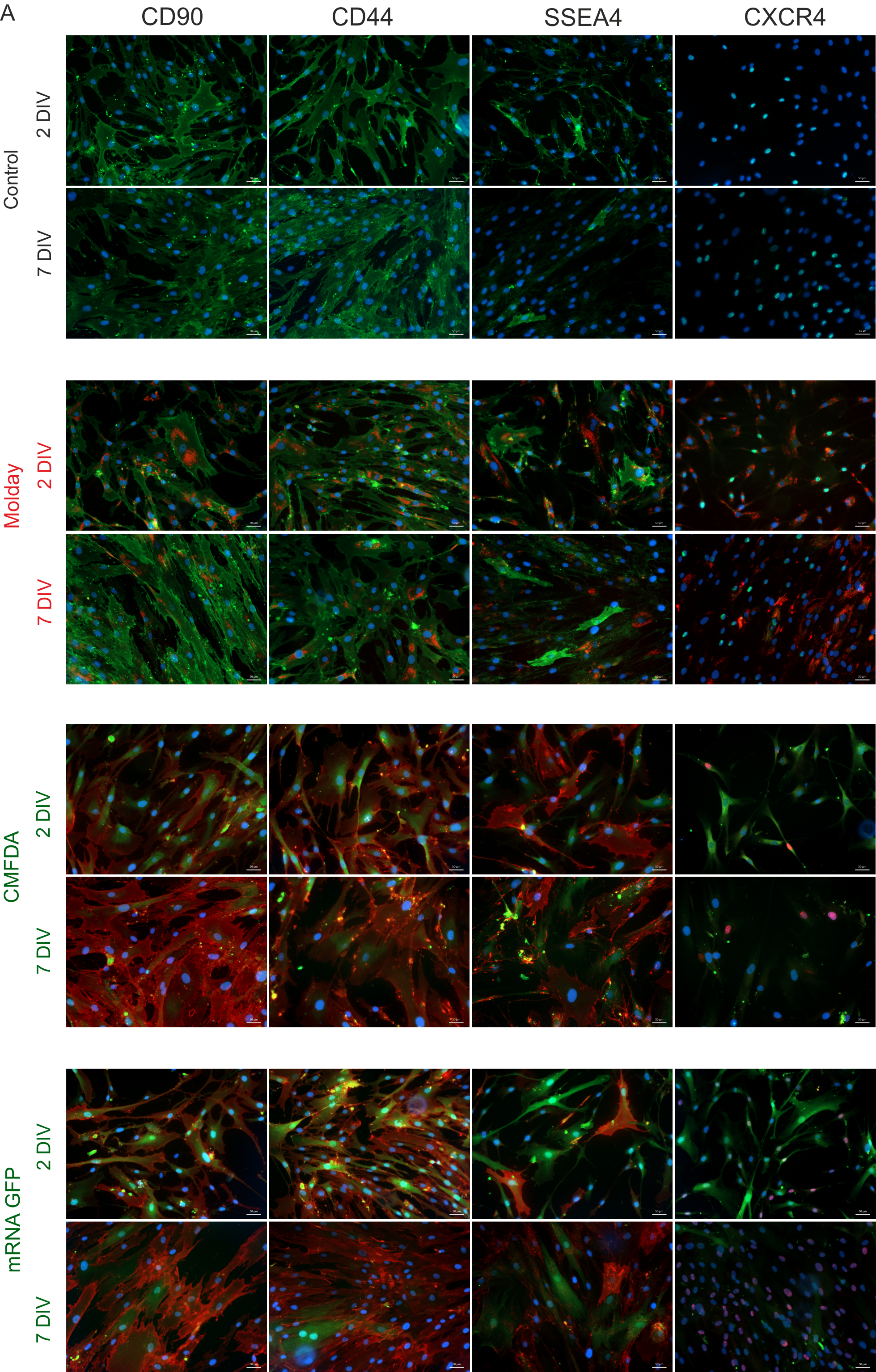

Supplement: Supplementary file 1 — Figure S1. Phenotypical analysis of hBM-MSC in the 2nd and 7th day of culture after staining with CellTracker™ Green CMFDA (CMFDA), eGFP (mRNA GFP), and Molday ION Rhodamine B™ (Molday) with antibodies directed against proteins: CD90, CD44, SSEA4, and CXCR4 performed by immunocytochemistry. Scale 50 μm. (PNG 5939 kb) [file 13287_2019_1296_MOESM1_ESM.png]

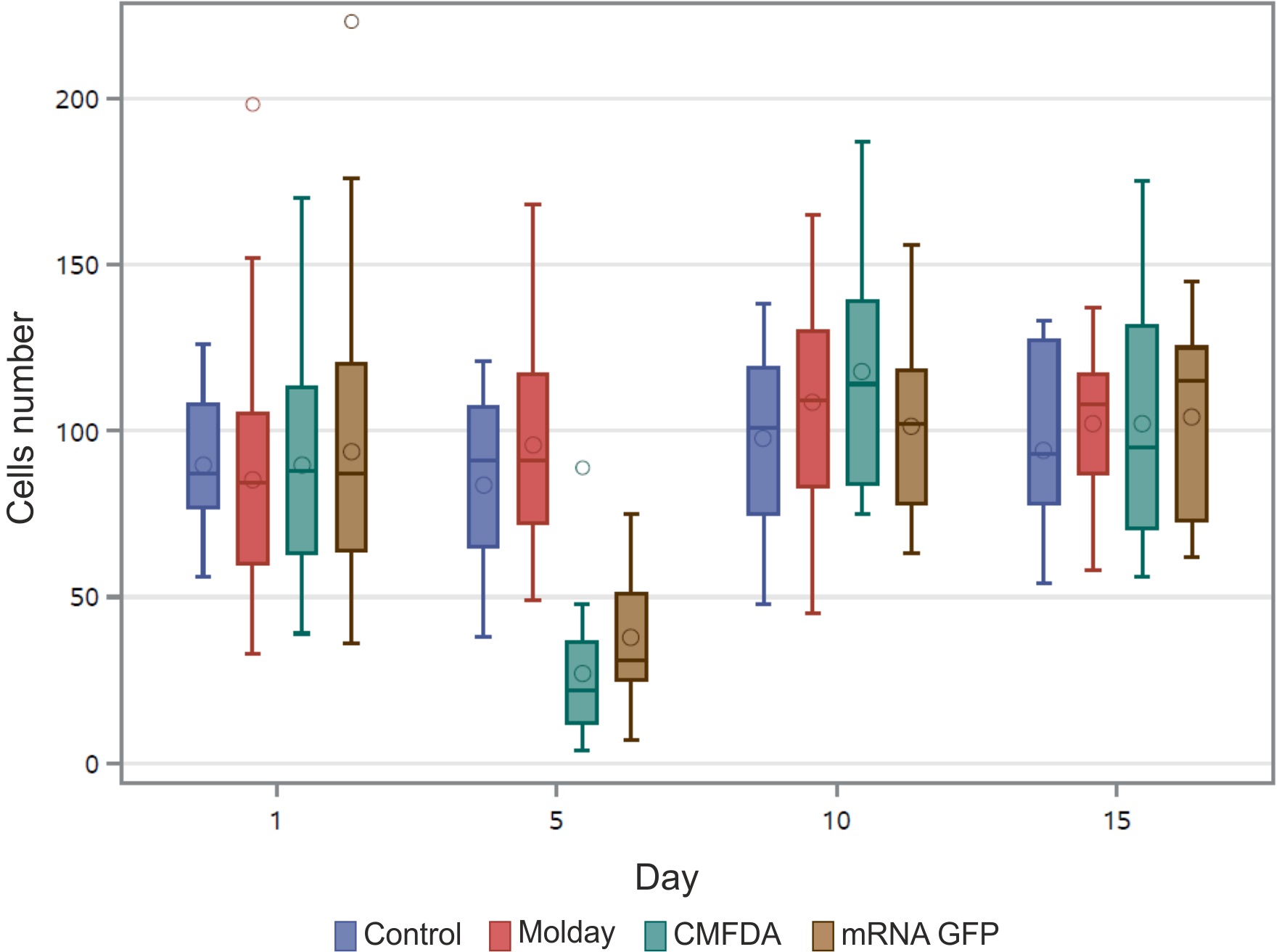

Supplement: Supplementary file 2 — Figure S2. The number of cells in particular groups during adipogenesis of hBM-MSC labeled with Molday ION Rhodamine B™ (Molday), CellTracker™ Green CMFDA (CMFDA), and eGFP (mRNA GFP). (JPG 178 kb) [file 13287_2019_1296_MOESM2_ESM.jpg]

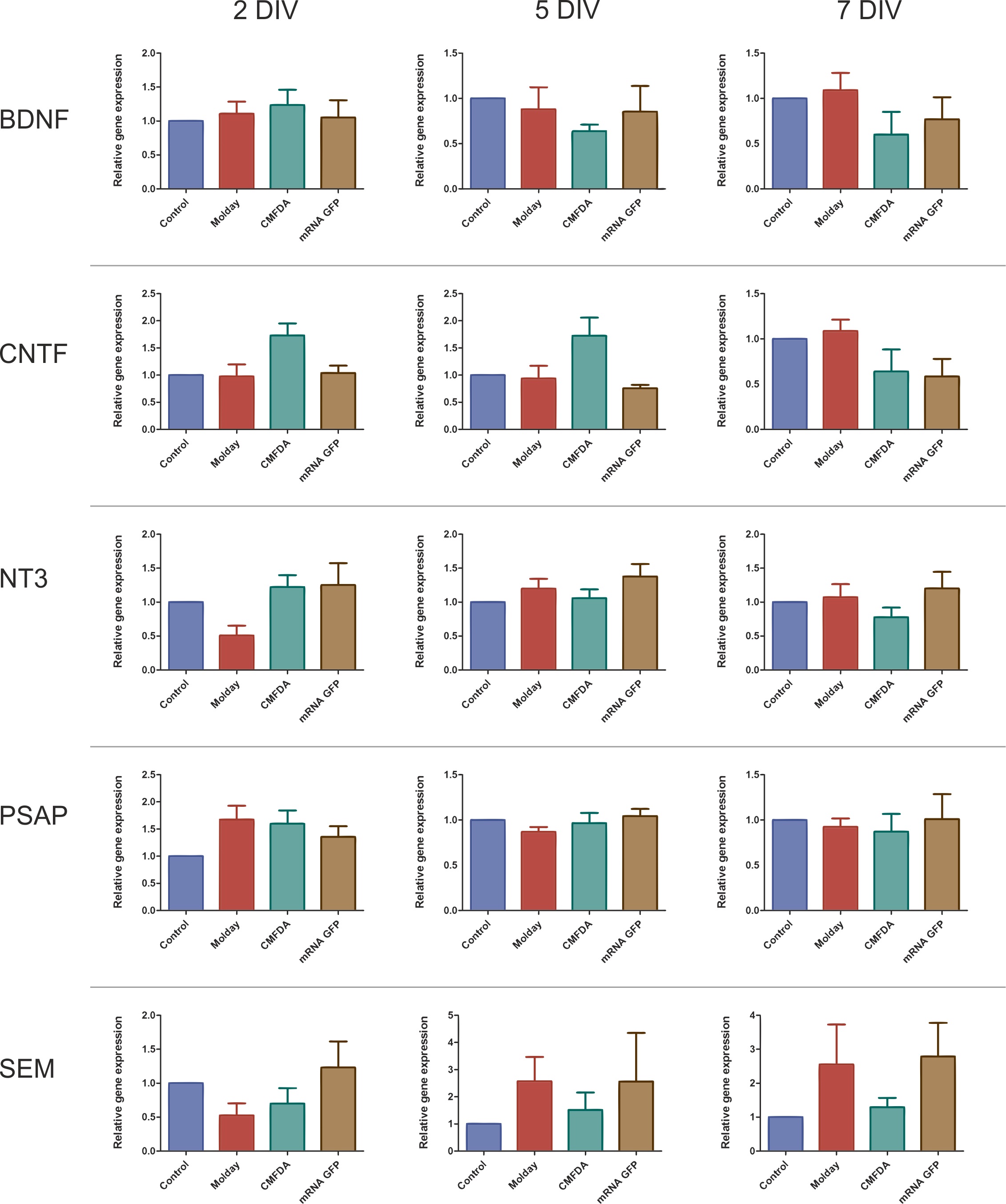

Supplement: Supplementary file 3 — Figure S3. Real-time PCR analysis of growth factors’ transcript level in cells stained with Molday ION Rhodamine B™ (Molday), CellTracker™ Green CMFDA (CMFDA), and mRNA eGFP (mRNA GFP) in comparison to unlabeled hBM-MSC in the 2nd, 5th, and 7th day after labeling, in which no statistically significant changes were observed. *p < 0.05, **p < 0.01, ***p < 0.001 (n = 5–7). (JPG 564 kb) [file 13287_2019_1296_MOESM3_ESM.jpg]
